# Cycling under the influence of alcohol-criminal offenses in a German metropolis

**DOI:** 10.1007/s00414-022-02828-8

**Published:** 2022-04-26

**Authors:** Jan-Bernd Bothorn, Holger Schwender, Matthias Graw, Peter Kienbaum, Benno Hartung

**Affiliations:** 1 Institute of Legal Medicine, University Hospital Düsseldorf, Düsseldorf, Germany; 2 Institute of Mathematics, Heinrich Heine University, Düsseldorf, Germany; 3 Institute of Legal Medicine, Ludwig Maximilians University, Munich, Germany; 4 Department of Anaesthesiology, University Hospital Düsseldorf, Düsseldorf, Germany

**Keywords:** Cycling under the influence, Alcohol, CUI, Criminal offense, Hipflask defence

## Abstract

**Introduction:**

Real or simulated cycling tests under the influence of alcohol might be biased by laboratory settings. Accident analyses consider incidents with injuries only. Herein, criminal offenses consisting of drunk cycling are evaluated in detail to fill this gap.

**Material and methods:**

All police-recorded cases of cycling under the influence of alcohol that took place in Düsseldorf, Germany, from 2009 to 2018 were identified. A total of 388 respective prosecutor’s files were available for analyses.

**Results:**

Mean blood alcohol concentrations were approximately 2 g/kg in both men and women. Men were overrepresented (6:1). Almost 60% of the cases were recorded between Friday and Sunday (the “weekend”). The average blood alcohol concentration (BAC) at night (01:00–05:59) was 0.39 g/kg lower than that during the day (06:00–17:59). Drinking after cycling allegations appear almost irrelevant among (German) cyclists. On average, the legal outcomes show 33 daily rates (median: 30). Additionally, the presented data raise doubts about whether the utilized medical tests or the ways in which they are carried out reliably discriminate between different grades of intoxication. Negative tests did not exclude high BACs, nor did positive tests correlate well with BACs.

**Discussion/Conclusion:**

In practice, CUI is seen with BACs above 1.60 g/kg in most cases. BACs below 1.60 g/kg either seem to be a minor problem or they have been incompletely addressed thus far. In summary, to be prosecuted, drunk cyclists have to ride their bikes in either a highly insecure or rude manner or they must cause an accident.

**Supplementary Information:**

The online version contains supplementary material available at 10.1007/s00414-022-02828-8.

## Introduction

It is estimated that more than 20% of road casualties are alcohol-related, even though the precise numbers vary from country to country, with a broad range from approximately 5 to 35%. This number corroborates with more than 250,000 deaths from alcohol-related road injuries worldwide every year [1].

In 2018, 4.4% of all cyclists involved in accidents on German roads were riding under the influence of alcohol. This number corresponds to 4236 cases and is well above the average of 2.4% for all road user groups [2]. Evaluations of bicycle-related crashes have found that more than one third of all injured persons were intoxicated with BACs above 1.60 g/l [3]. Bicycle or pedelec riders involved in road traffic accidents tend to sustain more severe injuries than those of motorised road users due to their lower mass and their lack of ability to dissipate collision energy [4]. This information underlines the need to ride a bicycle in a safe, unimpaired manner, which does not necessarily include only the state of soberness.

European legal limits vary considerably [5], and country-specific peculiarities exist. In Germany, if a BAC of at least 0.30 g/kg (g alcohol/kg blood) is present at the time of driving a car or riding a bicycle, then a criminal offense citation may be given if traffic-/medically relevant alcohol-related deficiency symptoms are detected (it is a so-called “relative impairment” to ride and/or drive while “under the influence of alcohol”), e.g. serpentine riding. In any case, a crime is committed on a bicycle or pedelec if a BAC of at least 1.60 g/kg is present at the time of riding (it is a so-called “absolute impairment” to ride a bicycle while under the influence of alcohol) [6]. This threshold is based on experimental studies by Schewe et al. [7, 8].

The different thresholds for “absolute impaired” riding for cyclists (1.60 g/kg) and “absolute impaired” driving for motor-vehicle users (1.10 g/kg) in Germany may be explained by the easier handling of a bicycle and the cyclist’s predominant risk of self-injury, which are factors that remain in the foreground. A study that examined the threshold for the absolute inability to ride a bicycle [9] demonstrated that at BACs of 1.40 g/kg, no test person was able to achieve the same riding performance that he or she displayed while sober. However, a few test subjects achieved performances on the cycling course at more than 1.60 g/kg that were comparable to other cyclists’ sober riding performances.

Since every real-cycling or real-driving study has methodological shortcomings (i.e. mainly highly motivated, healthy, and young test persons who are clearly aware of the test situation), it seems necessary to widen the scope to include data from everyday road traffic cases in which persons were actually cycling under the influence of alcohol (CUI). This can be achieved by analysing data from Düsseldorf, which is the capital of North Rhine-Westphalia.

North Rhine-Westphalia is the most populous German federal state. Düsseldorf has approximately 640,000 inhabitants [10] and is the seventh largest German city by population. Düsseldorf’s old town is known for its large number of pubs and nightlife options and is therefore a focal point for the consumption of alcoholic beverages.

In 2018, there were 358,154 households with an average size of 1.8 persons [11]. Even without taking into account the increasing availability of bike-sharing services, there were 1.7 bicycles per household. A total of 27.5% of households do not own a car, 47% of all citizens of Düsseldorf use a bicycle at least once a week, and 19% even daily or almost daily [12].

The hypotheses for the present work were as follows:

First, we assumed that intoxicated cyclists with comparable BACs are more often overstressed in real road traffic settings than in laboratory settings.

Second, we assumed that cyclists regularly show signs of impairment at BACs below 1.60 g/kg.

Third, as CUI is a criminal offense, which is why a medical examination is compulsory, the benefit of accompanying medical test results for impairment evaluation should be scrutinised.

These hypotheses were investigated by analysing public prosecutor files from the entire Düsseldorf city area over a period of 10 years.

The project was approved by the ethics committee of the University of Düsseldorf (study number: 2018–147-RetroDEuA), by the Ministry of the Interior of North Rhine-Westphalia and by the public prosecutor's office Düsseldorf.

## Material and methods

The period under review ranged from 2009 until 2018 (10 years) to avoid a lack of files due to ongoing cases. Pandemic-associated changes in traffic behaviour were not present in the given study period.

All police-recorded cases of CUI were identified by the Düsseldorf traffic police office after consent from the Ministry of the Interior of North Rhine-Westphalia. File numbers were transmitted to the Düsseldorf public prosecutor’s office (*N* = 637), where the respective files were identified.

A total of 249 case files were not available for data extraction, which led to a total of 388 analysable files. The following are possible reasons for the unavailability of files:

(a) Some files were in transit or had not yet been closed. In addition, revocation of the individual’s driver’s licence is often in question in cases of CUI, which would make the cases so-called urgent cases; this type of case could not be made available.

(b) The statute of limitations is 5 years, which is why some files had already been destroyed.

(c) In the case of defendants between the ages of 18 and 20, it is not the place of the criminal offense but rather the place of the individual’s residence that determines the jurisdiction of the public prosecutor's office; this is why some cases registered by the police had been transferred to other public prosecutor's offices.

CUI cases that included the additional consumption of substances other than alcohol were excluded (*N* = 16). Remarkably, forensic toxicological testing was carried out in only 3 of the remaining 372 cases.

Data of the available files (*N* = 372) were extracted by means of an evaluation matrix, which contained the following substantial points:

• File number
• Age and sex of the defendant
• Incident date and time
• Postal code of the defendant’s place of residence
• Postal code of the location of the incident
• BAC/BACs (in cases of alleged or possible drinking after cycling)
• Blood collection time
• Reason for police involvement
• Type of accident involvement
• Injury of the defendant or other involved persons
• Use of mobile phone without hands-free device
• Running through red lights
• Cycling without lights despite poor lighting conditions
• Insobriety symptoms with respect to alcohol-typical driving style
• Other special features, such as the court’s decision

The most essential components of each public prosecutor’s file were as follows:

• The police admission report
• Statements from the defendants and/or witnesses
• The medical report from the physician who took the blood sample (S1; **supplementary material**)
• The sheet for additional police findings from the standardised field sobriety test (so-called “Torkelbogen”)
• The report from the blood alcohol testing centre, including the determined blood alcohol concentration
• Correspondence from the defendant’s lawyer
• The verdict or penalty order

The medical report format used in North Rhine-Westphalia (S1; **supplementary material**) contains data such as body weight and constitution, previous illnesses and medication, blood loss and time of blood collection, which in certain cases allows for a back-calculation of the blood alcohol content or a better classification of the examination results. Furthermore, the medical tests prescribed by the document, which are mostly common in clinical neurological examinations, are described below, some of which require the cooperation of the person to be examined and are not obligatory for the test persons:

 − Gait: The test person is asked to walk approximately 10–15 steps straight ahead. Deviations to the side, swaying and a loss of balance are assessed [13].
 − Sudden turnaround: Following walking straight ahead, the subject is asked to make a sudden turn on the heel, and the examiner assesses the safe or unsafe execution [14].
 − Rotary nystagmus: The test person is rotated around his or her own axis 5 times within 10 s and then asked to fixate on a fixed point in the room. When sober, the resulting trembling is rather fine and only lasts for a short time. In the case of considerable intoxication, one expects a longer lasting (> 6 s) and coarser tremor, which cannot be influenced by the test person. The examiner documents the length of the eye tremor in seconds, as well as the speed (fast/slow) and the type (coarse/fine) of the deflection [15].
 − Finger-finger test: With the eyes closed, the test person brings the tips of their outstretched index fingers together in a lunging horizontal movement. Rough deviations are interpreted by the examiner as unsafe executions [13].
 − Finger-nose test: The tip of the index finger of an arm initially stretched out to the side is to be brought in an arc towards the tip of the nose. Rough deviations are judged by the examiner as unsafe executions [13].
 − Pupils: The width of the subject's pupils is assessed under normal room lighting without directly illuminating the eye. The standard values for pupil width vary interindividually, depend on age, and range between 4 and 9 mm. Any inconspicuous, dilated or constricted pupil width is documented [16].
 − Pupillary response to light: The examiner illuminates both eyes separately from the side and observes both the reaction of the pupil of the illuminated eye and the pupil of the nonilluminated eye. Prompt, delayed, or absent pupil responses are evaluated [16].

The sheet for additional police findings in the standardised field sobriety tests (so-called “Torkelbogen”) must be regarded as an amendment to the medical report. The basic tests are similar. However, it must be kept in mind that the police are in contact with the defendant approximately 30 min–1.5 h before the examining doctor gains contact with them. As this sheet was only partially filled out in most cases, it was not taken into account in the current study.

### Statistics

Mean values, medians and standard deviations were calculated for continuous variables.

Group comparisons were outlined by bar diagrams.

When considering continuous data, Student’s *t* test was used to test for differences in the mean between different groups, where a level of significance of *p* = 0.05 was considered.

Pearson’s correlation coefficient was calculated to measure the linear correlations between two variables.

To subanalyse the dataset, four age groups were defined. Age group 1 contained subjects below 30 years of age, age group 2 contained subjects from 30 to 39 years of age, age group 3 contained subjects from 40 to 49 years of age, and age group 4 contained subjects aged 50 years or more.

### BACs

The blood samples of all included files were analysed at the Institute of Legal Medicine in Düsseldorf according to German forensic guidelines [17].

## Results

A total of 372 cases were evaluated. The defendants were between 17 and 77 years old at the time of the incident (mean: 41.4; median: 40.5). A total of 319 defendants were male (mean age: 41.6), and 53 were female (mean age: 40.4).

### Forensically determined BACs

The detected BACs showed a range between 1.02 and 4.18 g/kg (S2; **supplementary material**); the median BAC was 1.94 g/kg.

In 61 cases, the BAC was below the absolute limit for impaired riding, i.e. 1.60 g/kg. A total of 311 cases were above or exactly at this value.

### Gender-specific differences in the BACs

**Fig. 1:**
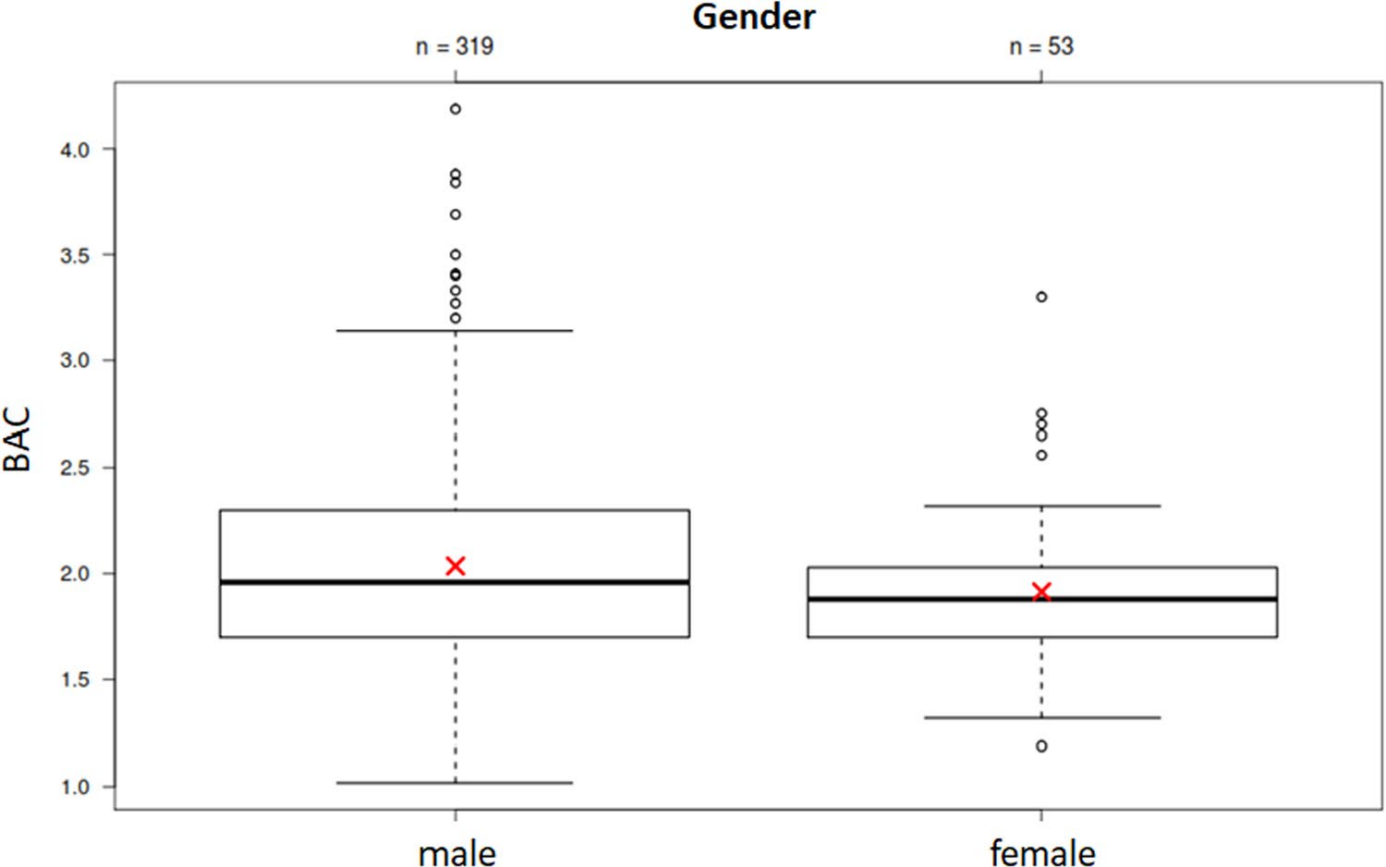
BAC comparison of males (*N* = 319) and females (*N* = 53; y-axis). The boxes contain 50% of the observations, the line inside the box indicates the respective median, the red crosses indicate average values and the satellites and respective outliers indicate 25% of the tested observations

The BACs of male and female subjects did not differ significantly from each other (*p* = 0.09; Fig. 1), even though median and average BACs were slightly higher in men than in women (men: mean BAC 2.04 g/kg, median BAC 1.96 g/kg; women: mean BAC 1.92 g/kg, median BAC 1.88 g/kg).

### Age-specific differences in the BACs

The median and average BACs of the subjects under 30 years of age (*N* = 77) were significantly lower than those of the subjects from 30–39 years of age (*N* = 100; p < 0.01) and those of the subjects from 40–49 years of age (*N* = 97; *p* = 0.01); they were also lower than those of the subjects who were 50 years old or older (*N* = 98), although not significantly.

### Weekday-specific differences in the BACs

**Fig. 2:**
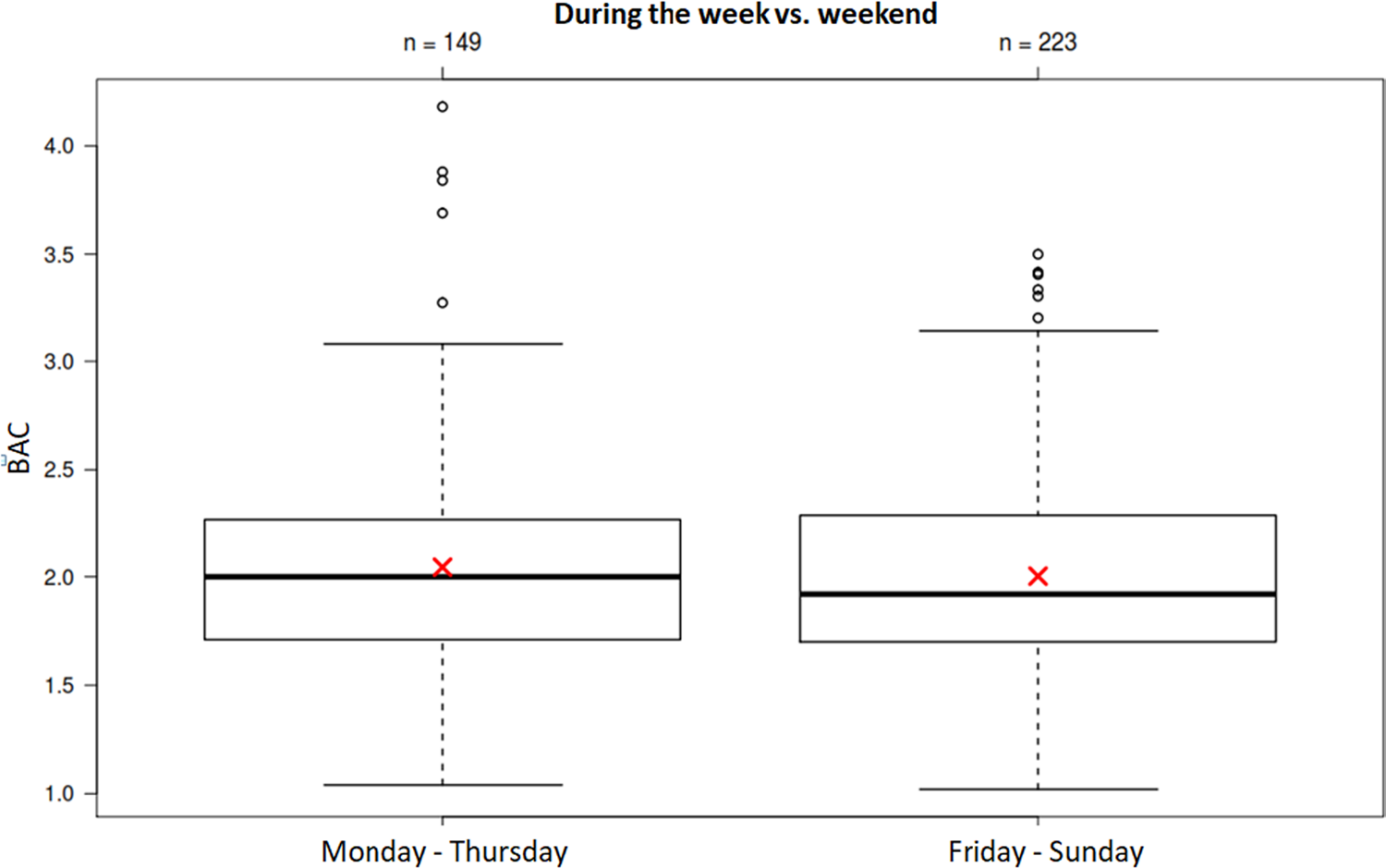
Comparison of defendants’ BACs (y-axis) during the “work week” (Monday–Thursday; *N* = 149) and the “weekend” (Friday–Sunday; *N* = 223)

Most cases (*N* = 223) were recorded from Friday until Sunday (the “weekend”, Friday *N* = 70, Saturday *N* = 73, Sunday *N* = 80), and 149 cases were recorded from Monday until Thursday (the “work week”; Monday *N* = 30, Tuesday *N* = 34, Wednesday *N* = 41, Thursday *N* = 44). A significant difference in the BAC levels between the “weekend” and the “work week” could not be determined (*p* = 0.43; Fig. 2).

### Daytime-specific differences in the BACs

When considering the subgroups of daytime (06:00–17:59; *N* = 65), early evening (18:00–21:59; *N* = 95), late evening (22:00–00:59; *N* = 99), and night-time (01:00–05:59; *N* = 113), BACs were significantly higher during the day (mean BAC 2.25 g/kg; median BAC 2.19 g/kg; range between 1.17 and 4.18 g/kg) than at night (mean BAC 1.86 g/kg; median BAC 1.81 g/kg; range between 1.31 and 2.80 g/kg) (*p* < 0.01).

### Drinking after cycling allegations

In only 14 cases, two blood samples were taken at different times, as drinking after cycling could not be ruled out. In 9 of these 14 cases, the second blood sample was ordered because the defendant had been unobserved between the alleged offense and the arrival of the police. In two of the 14 cases, the defendant had no recollection of whether he or she might have consumed alcoholic beverages after the alleged offense. In one of these two cases, a BAC of 3.08 g/kg was recorded in the first blood sample, and a BAC of 2.94 g/kg was recorded in the second blood sample. The time interval between the incident time and the first blood draw was 89 min. In the other case in which the defendant was unsure whether he or she had consumed further alcohol after the incident, a BAC of 2.30 g/kg was recorded in the first blood sample, and a BAC of 2.18 g/kg was recorded in the second blood sample. There was a span of 13 min between the incident and the first blood draw. In 1 of the 14 cases, the defendant drank from a plastic bottle in the patrol car on the way to the police doctor, and it could not be determined with certainty whether it was an alcoholic drink. In the 2 missing cases, the reasons for the second blood sample are not evident from the files.

In none of the 14 cases did the defendants claim to have been drinking after cycling, and in no case was the BAC of the second blood sample higher than that of the first blood sample.

### Reasons for police control

A total of 128 suspects came to the direct attention of the police, while in 15 cases, emergency services informed the police and in 9 cases, the public order office (“Ordnungsamt”) informed the police. Witnesses alerted the police in 126 cases. The process of police involvement was not specified in 94 cases.

In 133 cases, the cyclist’s conspicuous driving led to the immediate or delayed involvement of the police; in 78 cases, the cyclist was involved in a single accident, and in 110 cases, a traffic accident with another traffic participant was the reason for police involvement. The reason why police were involved was not specified in 51 cases.

### Driving conspicuities and traffic violations

A total of 104 cyclists were riding in a serpentine manner. Another 11 cyclists rode against the prescribed direction of travel, and 48 rode on the sidewalk without permission. The prohibited use of a mobile phone without a hands-free device was documented in 4 case files. There were 35 red light violations and 50 cases of driving without bike lights despite poor visibility. In cases where a red light was run, the BACs were significantly lower than those in cases where no red light violation was noted (*p* = 0.04). In cases of riding without lights despite poor light conditions, the BACs were significantly lower than those in cases where this issue was not noticed (*p* = 0.03).

### Conspicuities of the defendants during the medical examinations (carried out by a police physician)

**Fig. 3:**
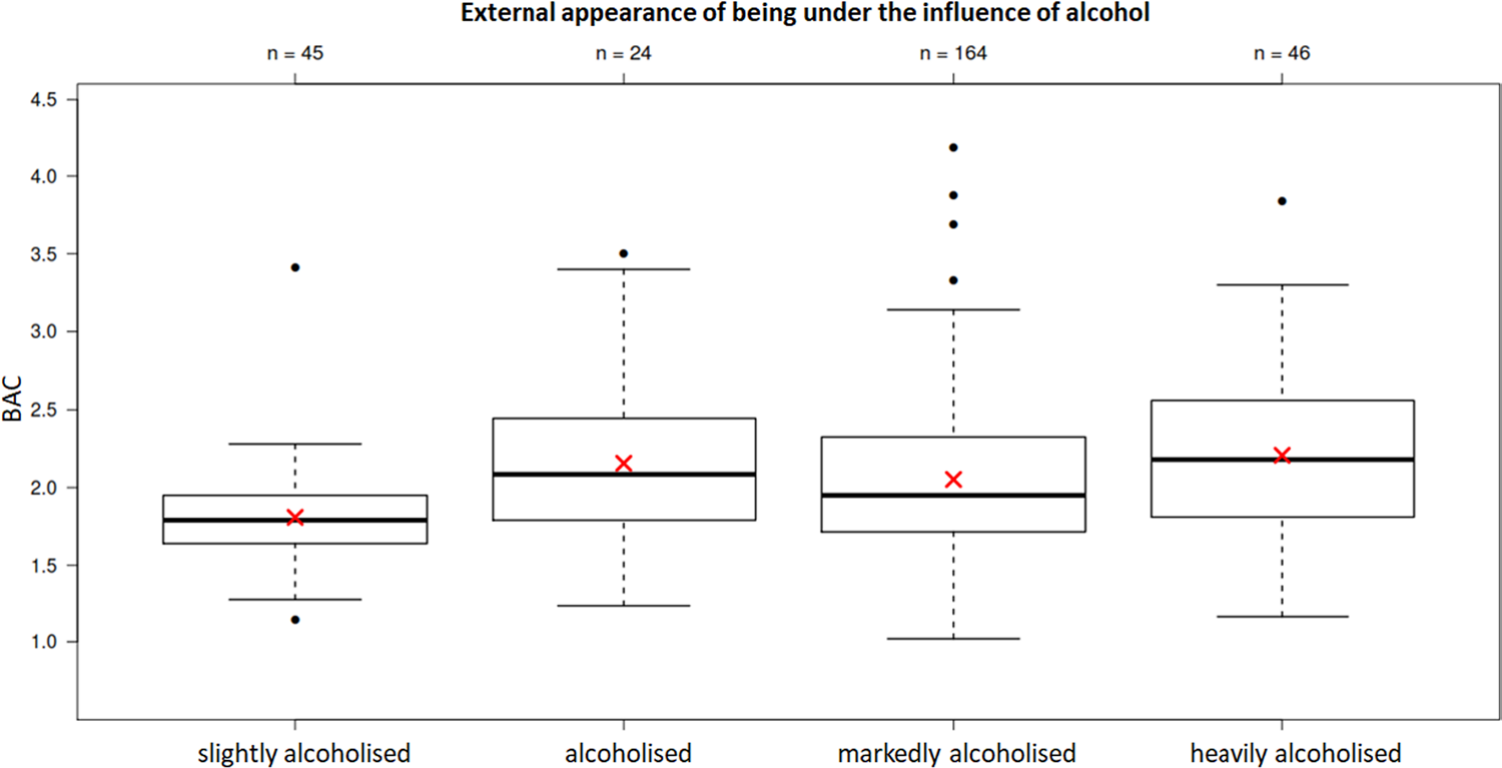
Comparison of defendants’ BACs (y-axis) with the external appearance of being under the influence of alcohol (“slightly influenced”/*N* = 45, “influenced”/*N* = 24, “markedly influenced”/*N* = 164, “heavily influenced”/*N* = 46)

• Blood sampling and examining physicians
  A medical report written by the physician who took the blood sample is available for 292 cases. At least 43 different physicians issued these medical reports. In addition, there were 33 medical reports that were neither stamped nor legibly signed. Six physicians conducted over 20 blood draws. Six other physicians performed between 6 and 16 blood draws. The remaining 31 distinguishable physicians each performed one or two blood draws.
• Gait
  Eighty-four persons showed a safe gait pattern and 135 persons showed an unsafe (dragging; swaying; staggering) gait pattern. This item was not specified for 153 persons.
  The test of making a sudden turn after previously walking in a straight line was also only partially carried out by the subjects. It was performed safely by 56 persons and unsafely by 57 persons. It was not documented in 259 subjects.
  An unsafe gait was seen at BACs of 1.02 g/kg or more. In an isolated case, a safe gait was seen at a BAC of 2.95 g/kg (S3; **supplementary material**). The BACs of persons with unsafe gaits were significantly higher than those of persons with safe gaits (*p* < 0.01) (S3; **supplementary material**). The BACs of subjects who conducted an unsafe turnaround were significantly higher than those of subjects who conducted a safe turnaround after previously walking in a straight line (*p* = 0.04; S4; **supplementary material**).
• Speech
  Clear speech was documented in 96 defendants, and slurred speech (to varying degrees) was documented in 170 defendants (not specified in 106 subjects). Slurred speech was seen in some cases starting from a BAC of 1.02 g/kg. Clear speech was seen in some cases up to a BAC of 4.18 g/kg. The median BAC was significantly higher for those with slurred speech than for those with clear speech (p < 0.01) (S5; **supplementary material**).
• Finger coordination tests
  The so-called finger-finger test was performed safely by 72 persons and unsafely by 76 persons (S6; **supplementary material**). It was not specified in 224 subjects. The BACs of subjects with safe and unsafe finger-to-finger tests did not differ significantly from each other (*p* = 0.09).
  The finger-nose test was performed safely by 90 subjects and unsafely by 55 subjects (S7; **supplementary material**). It was not specified in 227 subjects. Thereby, the defendants’ BACs were significantly higher if unsafe finger-nose tests were evident (*p* < 0.01).
• Consciousness
  Full consciousness was noted in 193 subjects, while in 73 subjects, documentation showed dazed and/or confused consciousness (S8; **supplementary material**). Consciousness was not specified in 106 cases. The BACs of subjects with nonclear consciousness (mean = 2.18, median = 2.12, SD = 0.49) were significantly (*p* < 0.01) higher than those of subjects with clear consciousness (mean = 1.97 median = 1.88, SD = 0.52).
• External appearance under the influence of alcohol
  There was no external appearance of being under the influence of alcohol in 5 defendants. Forty-five defendants appeared slightly under the influence of alcohol, 164 appeared markedly, and 46 appeared either heavily or very heavily under the influence of alcohol. Twenty-four defendants appeared influenced by alcohol without any further classification of markedness (Fig. 3). This item was not specified in 88 subjects. There were significant differences of the BACs in persons with different external appearances of being under the influence of alcohol (*p* < 0.01; one-way ANOVA). The BAC of persons with slight external appearance of being under the influence of alcohol was significantly lower than those with markedly or heavily alcohol-intoxicated appearance.
  There were no significant differences between males and females (*p* = 0.12) regarding the external appearance of being under the influence of alcohol.

• Abnormalities of the eyes
  The pupil size of 154 subjects appeared inconspicuous. Six subjects had conspicuously constricted pupils, and 99 subjects had dilated pupils (S9; **supplementary material**). This finding was not specified in 113 subjects. The pupillary response to light was prompt in 106 subjects, delayed in 114 subjects, and absent in 12 subjects. This finding was not specified in 140 subjects.
  Subjects with an inconspicuous pupil size had significantly higher BACs compared with subjects with dilated pupils (*p* = 0.04).
  The BACs of subjects with a prompt pupil response (mean = 2.03; median = 1.92) were not significantly different from the BACs of subjects with a delayed pupil response (mean = 2.01; median = 1.93) (*p* = 0.90). The subgroup of those without a pupil response was too small for separate consideration (*N* = 12) (S10; **supplementary material**).
• Testing of the rotary nystagmus
  Coarse-beat rotary nystagmus was seen in 19 subjects, and fine-beat rotary nystagmus was seen in 63 subjects. In 7 further cases, both coarse-beat and fine-beat rotary nystagmus were documented at the same time. This item was not documented in 283 cases.
  The speed of deflection was reported as being slow in 9 cases and fast in 67 cases and was not reported in 296 cases.
  In 83 of the 372 cases, the duration of (any) rotary nystagmus was measured as ranging from 5 to 39 s, and the correlation to the BAC was poor (Pearson’s correlation coefficient: 0.28, p: 0.01). The measured duration of (any) rotary nystagmus was significantly lower in subjects with a (slight) outward appearance of being under the influence of alcohol than in subjects with a marked outward appearance of being under the influence of alcohol (*p* = 0.02).

### Legal outcomes

**Fig. 4:**
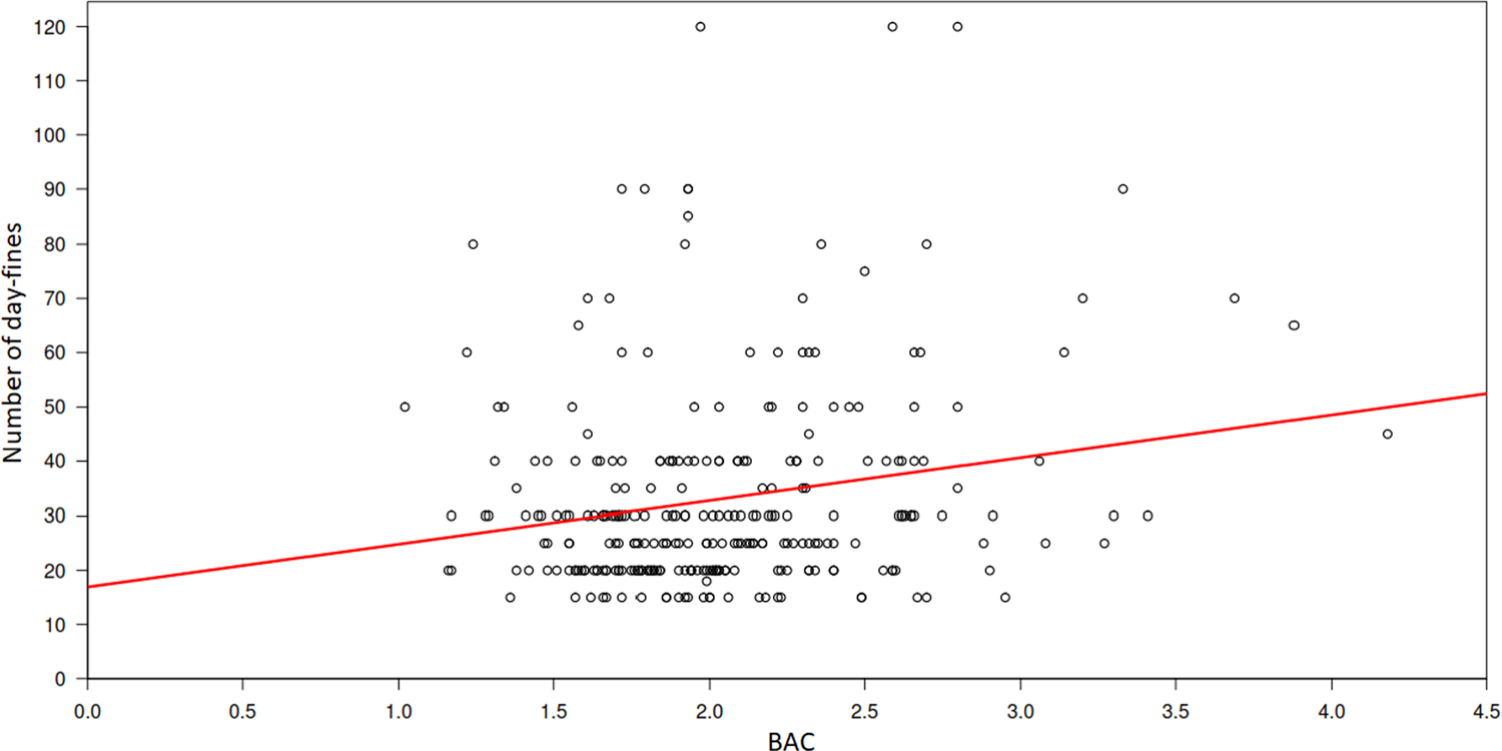
Correlation between BACs (x-axis) and day fines (y-axis)

A total of 282 cases resulted in fines or penalties ranging between 15 and 120 day-fines (“Tagessatz”, a fine calculated on the daily rate of income) (mean: 33 day-fines, median: 30 day-fines) between 5 and 100 € (mean: 30 €, median: 30 €). Five proceedings resulted in a custodial sentence due to relevant and/or multiple previous convictions, 4 of which were suspended for probation. Seventy-seven proceedings were discontinued, 11 of which were discontinued due to the unknown whereabouts of the defendant, 8 were discontinued because penalties in other proceedings against the defendant prevailed, and 36 proceedings were discontinued after one-time payments were made to the state treasury or a charitable institution. After the death of one defendant and after the deportation of another defendant to his home country, each of these proceedings was discontinued. In 20 further cases, the reason for discontinuation was not reported. In 8 files, the legal outcome was not reported. In the cases that resulted in fines/penalties, the level of BAC had a significant impact on the number of day-fines (*r* = 0.20; *p* < 0.01; Fig. 4).

### Correlation between the reason for the control and the amount of penalty

When considering the reason for police control, traffic accidents led to significantly higher numbers of day fines than did cases with unsafe (conspicuous) cycling alone (*p* < 0.01). The number of day fines between traffic accidents with other traffic participants and single bicycle accidents (as a reason for control) did not differ significantly (*p* = 0.13) nor did the differences between single accidents and cases of unsafe conspicuous cycling (*p* = 0.84).

### Separate consideration of the cases of relative driving unsafety

Sixty-one cases were seen with BACs below the current limit of absolute impaired driving in Germany of 1.60 g/kg (mean: 1.41 g/kg; median: 1.45 g/kg). Among them were 9 (14.8%) female and 52 (85.2%) male defendants.

**Fig. 5:**
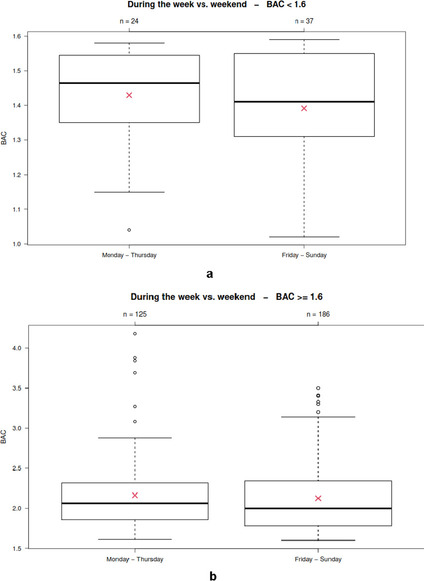
**a**. Comparison of BACs (y-axis) in defendants with BACs below 1.60 g/kg during the “work week” (Monday–Thursday; *N* = 24) and the “weekend” (Friday–Sunday; *N* = 37). **b**. Comparison of BACs (y-axis) in defendants with BACs of at least 1.60 g/kg during the “work week” (Monday–Thursday; *N* = 125) and the “weekend” (Friday–Sunday; *N* = 186)

Most of the cases with BACs below 1.60 g/kg were recorded from Friday until Sunday (*N* = 37; 60.7% vs. 59.8% in cases ≥ 1.60 g/kg). A significant difference in the BAC levels between the “weekend” and the “work week” could not be determined (*p* = 0.35 vs. *p* = 0.46 in cases ≥ 1.60 g/kg; Fig. 5a and b).

There were 7 single accidents (11.5% vs. 22.8% in cases ≥ 1.60 g/kg), 27 traffic accidents (44.3% vs. 26.7% in cases ≥ 1.60 g/kg), 26 cases of conspicuous cycling (42.6% vs. 34.1% in cases ≥ 1.60 g/kg) as cause for police control and one file without a reported type of incident (1.6% vs. 16.4% in cases ≥ 1.60 g/kg). In 19 of the 26 cases of conspicuous cycling (73.1% vs. 66% in cases ≥ 1.60 g/kg), cycling in a serpentine manner was the reason for police intervention.

**Fig. 6:**
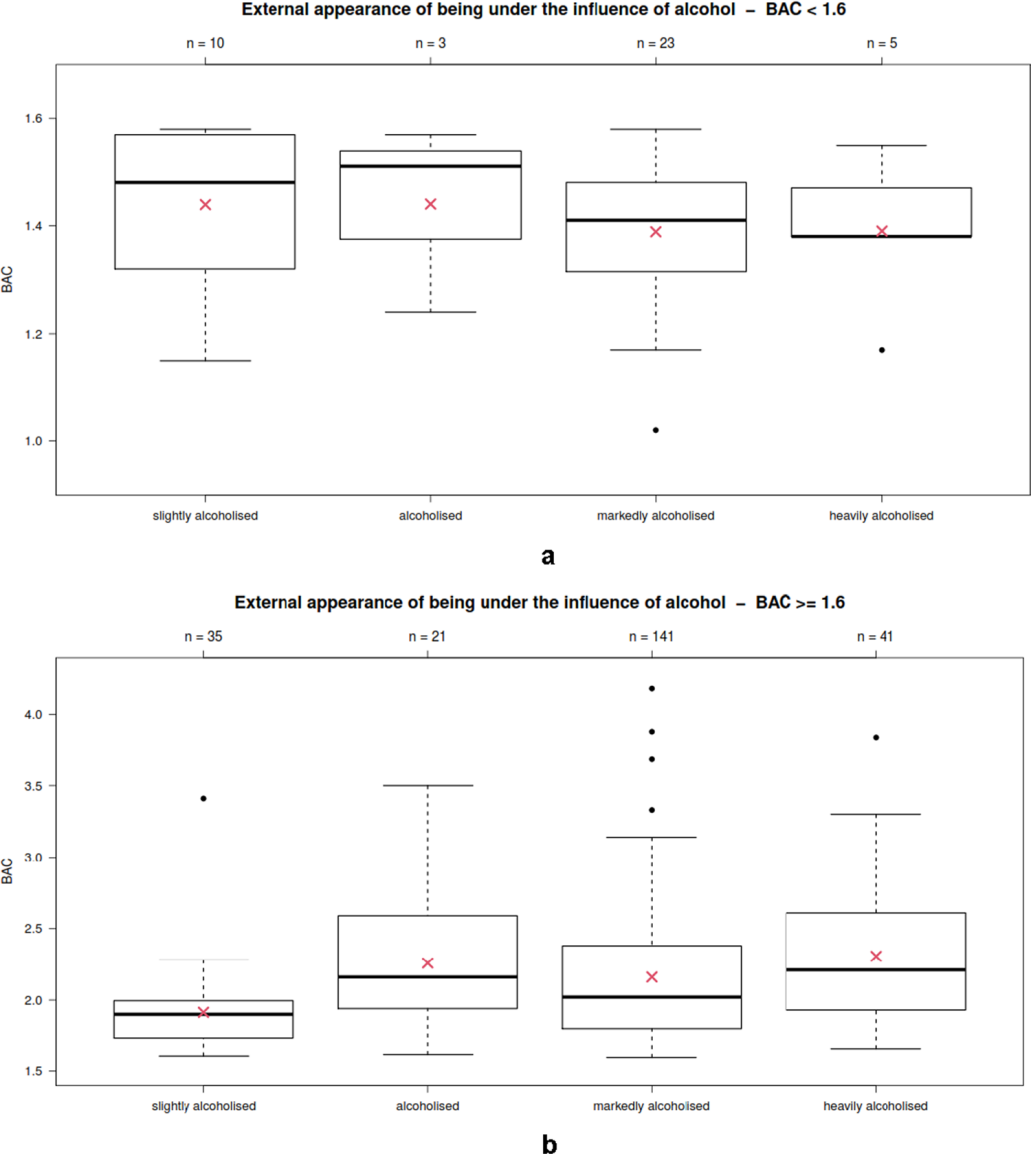
**a**. Comparison of BACs (y-axis) in defendants with BACs below 1.60 g/kg with the external appearance of being under the influence of alcohol (“slightly alcoholised”/*N* = 10, “alcoholised”/*N* = 3, “markedly alcoholised”/*N* = 23, “heavily alcoholised”/*N* = 5). **b**. Comparison of BACs (y-axis) in defendants with BACs of at least 1.60 g/kg with the external appearance of being under the influence of alcohol (“slightly alcoholised”/*N* = 35, “alcoholised”/*N* = 21, “markedly alcoholised”/*N* = 141, “heavily alcoholised”/*N* = 41)

In subjects with a BAC below 1.60 g/kg, there was no external appearance of being under the influence of alcohol in 2 (3.3% vs. 1% in cases ≥ 1.60 g/kg) defendants. Ten (16.4% vs. 11.3% in cases ≥ 1.60 g/kg) defendants appeared slightly alcohol-intoxicated, 23 (37.7% vs. 45% in cases ≥ 1.60 g/kg) appeared markedly alcohol-intoxicated, and 5 (8.2% vs. 13.2% in cases ≥ 1.60 g/kg) appeared heavily or very heavily under the influence of alcohol. In three cases (4.9% vs. 6.8% in cases ≥ 1.60 g/kg), the defendants appeared alcohol-intoxicated but there was no further classification of markedness available. In subjects with a BAC below 1.60 g/kg, the level of markedness was not specified in 18 (29.5% vs. 22.8% in cases ≥ 1.60 g/kg) cases (Fig. 6a and b).

Among the 61 cases with a BAC below 1.60 g/kg, there were 39 proceedings that ended in fines/penalties. In 9 of the 39 cases, the penalty orders/judgements were based on findings from the police doctors, in addition to the BAC and cycling incidents. In 30 cases, the fines/penalties were based on police findings or witness statements. Eighteen proceedings were discontinued, 10 of which were discontinued after a one-time payment was made to the state treasury or a nonprofit institution, and 4 of which were discontinued because the individual’s level of culpability was considered to be low. The reason for the discontinuation of the remaining 4 cases was not specified. In 4 of the 61 cases with a BAC below 1.60 g/kg, the result of the proceedings was not reported.

## Discussion

The method chosen for the current work was a retrospective analysis of prosecutorial files (real life cases) that aimed to expand the views of injury-focussed case–control studies [18–20] and laboratory cycling test findings [7–9, 21, 22]. Case–control studies in this area typically consider injured cyclists or those involved in alcohol-related accidents but not those cyclists with conspicuous alcohol-typical cycling.

Typical drunk cyclists are often said to be young male adults, which was confirmed herein (319 males, 53 females). The determined BACs were almost equally distributed between the sexes. Even though differences were not statistically significant, men showed slightly higher BACs than women in real-life situations (men: mean BAC 2.04 g/kg, median BAC 1.96 g/kg; women: mean BAC 1.92 g/kg, median BAC 1.88 g/kg), which is in line with the results of laboratory tests [21]. The average BAC at night (01:00–05:59) was 0.39 g/kg lower than that during the day (06:00–17:59). Only the subgroup below 30 years of age showed slightly lower BACs on average.

Almost 60% of the cases were recorded between Friday and Sunday (the “weekend”). Contrary to what one might expect, the BACs of the weekend cases did not differ significantly from those that occurred between Monday and Thursday. It seems that cyclists who are not severely impaired are only prosecuted if egregious traffic behaviour is evident (e.g. running red lights).

Drinking and cycling habits, cyclable distances and permissive attitudes are said to increase the probability of CUI [23]. Permissive attitudes were confirmed herein.

Surprisingly, drinking after cycling allegations, which are common among car drivers [24, 25], appear almost irrelevant among (German) cyclists. One reason for this could be the fact that a high number (N = 137) of defendants were road-side tested by the police or stopped by the public order office and thus were not unobserved at any time. For the remaining subjects, the presumably lower level of awareness of the impending penalties might be an explanation [23]. Furthermore, it can be hypothesised that some defendants do not fear the suspension of their driver’s licence because they mainly ride a bicycle anyway and/or do not have a driver’s licence. The judicial prohibition of riding a bicycle exists only very sporadically in Germany. The examined cyclists rarely took advantage of legal advice; thus, one could only speculate if legal advice leads to more hipflask defences.

The legal outcome on average was 33 daily rates (median: 30). The height of the daily rates was 30 € on average (median 30 €), which indicates an available average income of 900 € per month among the examined drunk cyclists. In general, the penalties increased with increasing BAC levels and the consequences of the driving behaviour.

The files also presented interesting insights into the results and performance of the accompanying medical tests. Evaluations should scrutinise the benefits of the accompanying medical test results for the interpretation of impaired cycling. However, the results are somewhat confounding and partially contradictory. When looking at the results of these medical tests, it generally has to be kept in mind that the identification of intoxication can be difficult for laypeople [26] and for experts [27]. Nevertheless, the examining physician is usually informed about preliminary breath alcohol results if they were performed (successfully).

In fact, all coordinative tests are based upon the cooperation of the defendant (finger-finger-test, finger-nose-test, gait, sudden turnaround after walking). Most of the tests examined herein showed only slightly more distinctive features at higher BACs, and some were hardly distinctive at all. For example, in the examination of gait, all the tests were conspicuous only at a blood alcohol concentration of more than 2.95 g/kg. When examining speech, it was noticeable that in the two cases with the highest BACs (3.88 g/kg and 4.18 g/kg), a clear pronunciation was documented. Similar results were obtained for finger-finger tests, finger-nose tests, and sudden turnaround after walking tests, where only very high BACs (above approx. 3 g/kg) led to insecure results in almost all cases.

Even though these coordinative tests are necessary to identify alcohol-related impairments, the presented data raise doubts about whether the medical tests or the ways in which they are carried out reliably discriminate between different grades of intoxication. Negative tests did not exclude high BACs, nor did positive tests correlate well with the BAC.

Non-coordinative medical tests, such as the coarse-beat rotary nystagmus, were also not able to clearly identify intoxicated persons. Even though the rotary nystagmus test has been shown to be a reliable test for examining intoxicated persons [28] with a sensitivity of 0.72 and a specificity of 0.78, this reliability could not be confirmed. We can only assume that this test was not carried out in the prescribed way in all cases.

Therefore, it must be pointed out that a synoptic evaluation of possibly riding a bicycle while impaired is inevitable. The manner in which the bike is ridden is crucial, as most of the medical tests—that are carried out with delay—do not always reliably indicate the grade of the influence of alcohol.

It is remarkable that only 61 of 372 subjects showed accusable BACs below 1.60 g/kg. This finding contradicts the first and second hypotheses that cyclists regularly show signs of impairment at BACs below 1.60 g/kg in real traffic situations. One possible explanation might be that cycling is a simple routine task that can be carried out safely even at higher BACs as long as no challenging situations occur. Another possible explanation might be that blood samples were obtained only if cyclists without noticeable driving impairments or accidents gave the police a reason to believe that they were severely influenced by alcohol. In addition to a noticeable smell of alcohol, this situation might have arisen especially in those cases where a preliminary breath test result indicated the equivalent of a BAC of significantly over 1 g/kg.

Furthermore, it cannot be excluded that some defendants were also under the influence of substances other than alcohol, as forensic toxicological testing was only carried out in 3 of 372 cases. Nevertheless, the influence of another substance other than alcohol should only lead to more impaired cyclists.

## Conclusions

In practice, CUI is seen at BACs above 1.60 g/kg in most cases. BACs below 1.60 g/kg either seem to be a minor problem or they have been incompletely addressed thus far.

In summary, in order to be prosecuted, drunk cyclists have to regularly ride their bikes in either a highly insecure or rude manner or they must cause an accident.

Finally, the results from standardised field sobriety tests must be interpreted synoptically.

## Limitations

The proportion of drunk cyclists who were not recorded by the police are not represented herein, which means that this proportion of the population cannot be addressed scientifically.

It was also not possible to analyse sober cyclists who cycled conspicuously and unsafely even though they were not intoxicated.

Selection bias due to the control behaviour of the police cannot be ruled out, e.g. with the BAC determination of severely impaired cyclists mainly. Furthermore, different control criteria being used at different times could be possible, e.g. due to certain checkpoint locations or time slots, the individual characteristics of the police officers in charge or the external appearance of the cyclists.

In only 3 of 372 cases, forensic toxicological testing was carried out. Therefore, it cannot be ruled out that some defendants were under the influence of substances other than alcohol.

Finally, 249 of 637 files were not available; reasons for this lack of availability included urgent cases, cases of defendants between 18 and 20 years of age who do not live in Düsseldorf and closed files that had exceeded the statute of limitations, all of which could not be considered in the current study.

## Supplementary Information

Below is the link to the electronic supplementary material.

Supplementary file1 (DOCX 475 KB)
