# Supplementary material for: Cycling under the influence of alcohol-criminal offenses in a German metropolis
Source: Int J Legal Med. 2022 Apr 26;136(4):1121–32. doi: 10.1007/s00414-022-02828-8 (PMC9170663; doi:10.1007/s00414-022-02828-8)
Supplement: Supplementary file 1 — Supplementary file1 (DOCX 475 KB) [file 414_2022_2828_MOESM1_ESM.docx]

**Figures, supplementary material:**

**S1; supplementary material:** Example of a medical report that includes a physical examination and documentation of the subjective findings in a standardised manner (translated to English).


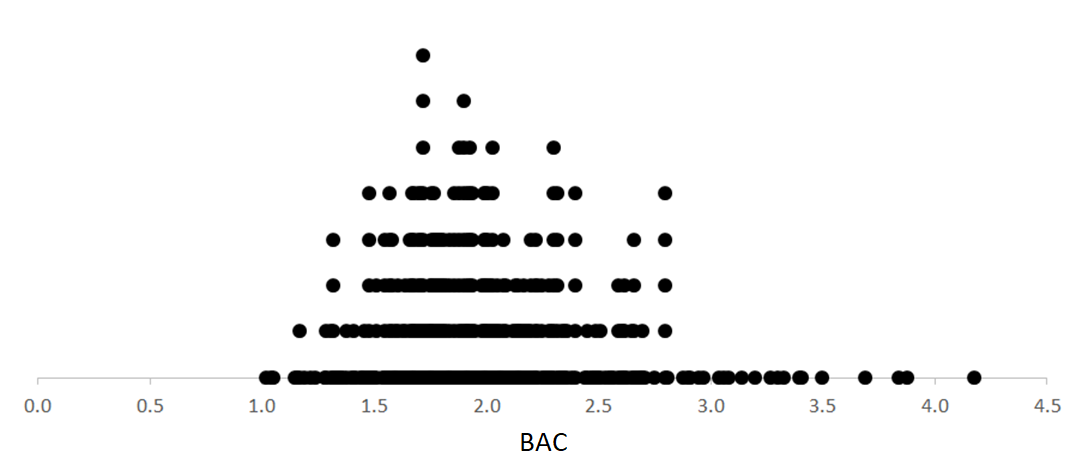
**S2; supplementary material:** Overview and frequencies of all measured BACs (N=372). Each measured BAC is represented by a dot. If the same BAC occurs more than once, additional dots are drawn above.


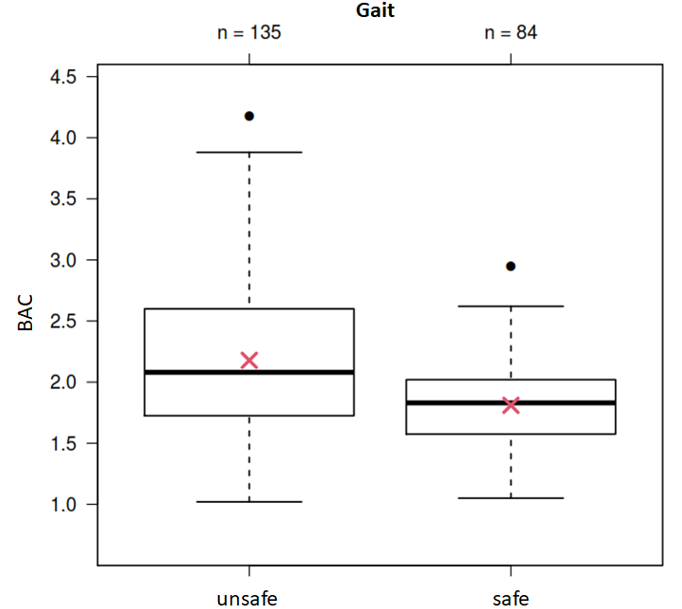


**S3**; **supplementary material:** Comparison of defendants’ BACs (y-axis) with “unsafe” (N=135) and “safe” (N=84) gaits.


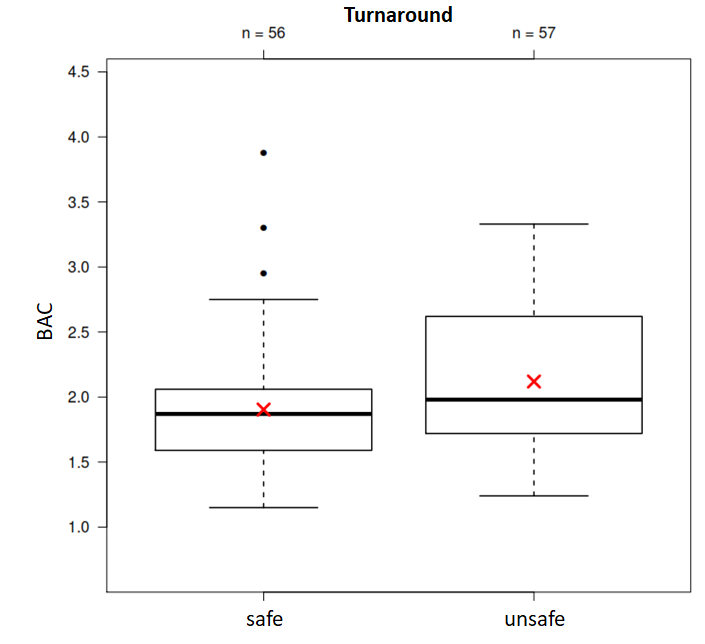


**S4**; **supplementary material:** Comparison of defendants’ BACs (y-axis) with “safe” (N=56) and “unsafe” (N=57) sudden turnaround while walking.


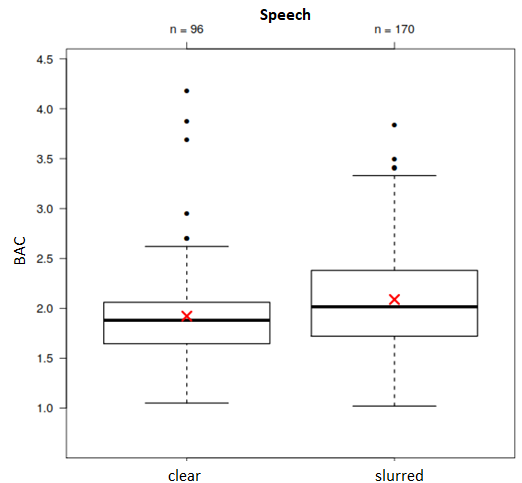


**S5**; **supplementary material:** Comparison of defendants’ BACs (y-axis) with “clear” (N=96) and “slurred” (N=170) speech.

**
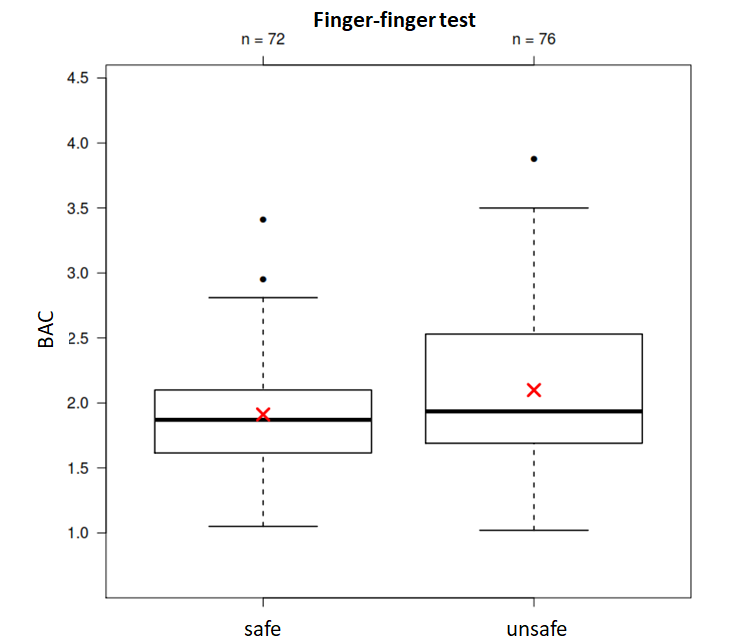
**

**S6**; **supplementary material:** Comparison of defendants’ BACs (y-axis) with “safe” (N=72) and “unsafe” (N=76) finger-finger tests.


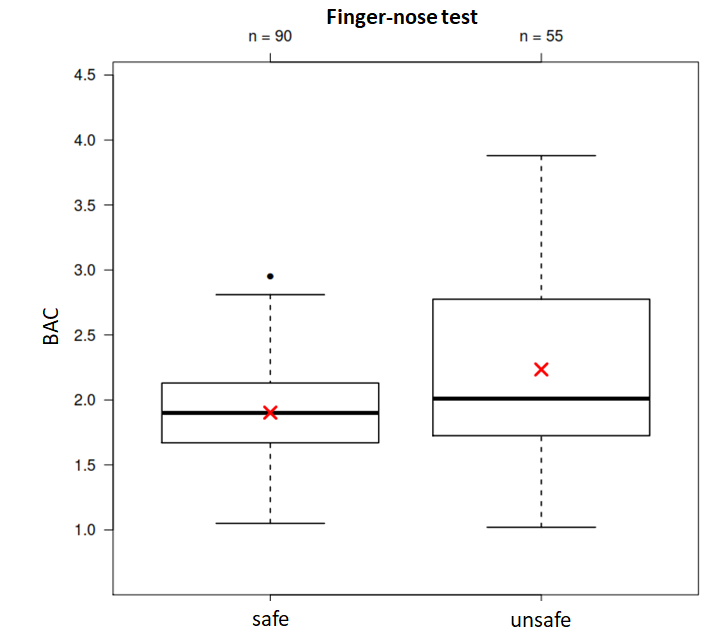


**S7**; **supplementary material:** Comparison of defendants’ BACs (y-axis) with “safe” (N=90) and “unsafe” (N=55) finger-nose tests.


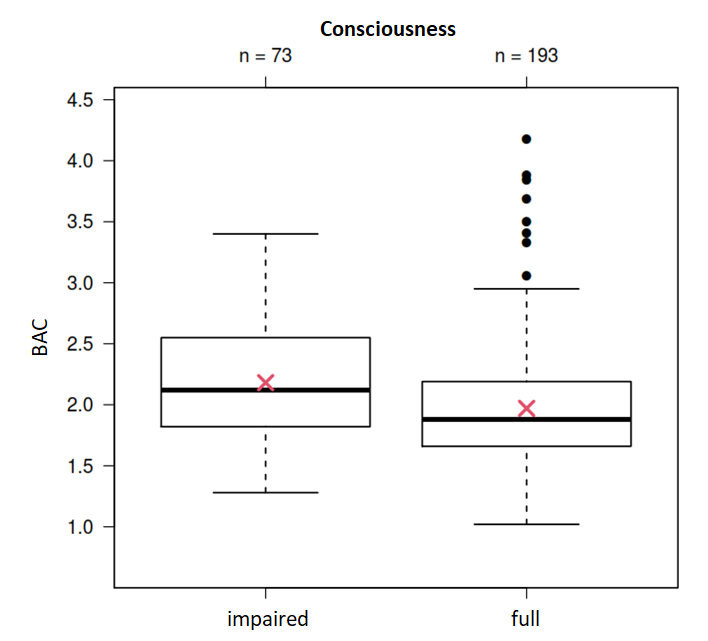


**S8**; **supplementary material:** Comparison of defendants’ BACs (y-axis) with “impaired” (N=73) and “full” (N=193) consciousness.


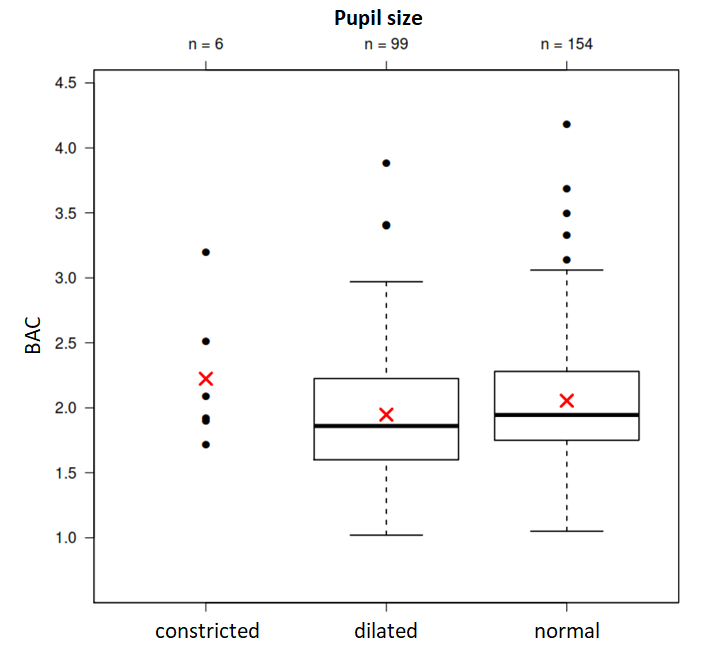


**S9**; **supplementary material:** Comparison of defendants’ BACs (y-axis) to constricted (N=6), dilated (N=99) and normal (N=154) pupil sizes.


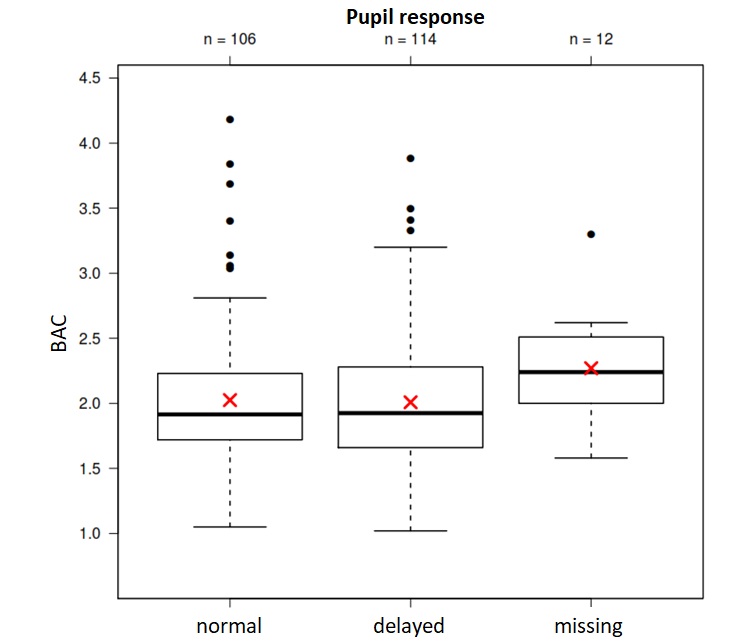


**S10**; **supplementary material:** Comparison of defendants’ BACs (y-axis) to normal (N=106), delayed (N=114) and missing (N=12) pupil responses.
